# Visceral Adiposity Is an Independent Determinant of Hypercoagulability as Measured by Thrombin Generation in Morbid Obesity

**DOI:** 10.1055/s-0037-1608942

**Published:** 2017-12-15

**Authors:** P. B. Chitongo, L. N. Roberts, L. Yang, R. K. Patel, R. Lyall, R. Luxton, S. J. B. Aylwin, R. Arya

**Affiliations:** 1 Department of Haematological Medicine, King's Thrombosis Centre, King's College Hospital, London, United Kingdom; 2 Department of Endocrinology, King's College Hospital, London, United Kingdom; 3 Department of Respiratory Medicine, Kings College Hospital, London, United Kingdom; 4 Faculty of Health and Applied Sciences, University of the West of England, Bristol, United Kingdom

**Keywords:** coagulation factors, visceral adipose tissue, hemostatic marker, tissue factor pathway inhibitor, venous thrombosis

## Abstract

**Introduction** Increased visceral adipose tissue (VAT) has been shown to be associated with the development of insulin resistance, type 2 diabetes, stroke, and ischemic heart disease. It remains unknown whether fat distribution impacts on coagulation markers and/or the risk of venous thrombosis. This study evaluates markers of hypercoagulability in **class** III obesity (body mass index [BMI] >40 kg/m ^2^ ) compared with nonobese controls. We further investigated whether hypercoagulability was influenced by VAT, metabolic syndrome, and metabolic markers, including adiponectin.

**Patients and Methods** Ninety patients were recruited from the obesity clinic at King's College Hospital from November 2009 to December 2011. The inclusion criteria were class III obesity (BMI ≥40 kg/m ^2^ ) and age 18 to 65 years. A control group (healthy ambulatory participants, with a BMI < 30 kg/m ^2^ ) was recruited from volunteers responding to advertisement. Abdominal VAT and subcutaneous adipose tissue surface areas were determined by evaluation of a single-slice CT at spinal vertebra L4.

**Results** Thrombin generation revealed a significantly increased peak and endogenous thrombin potential in patients compared with controls. Lag time and time to peak (ttP) were also significantly prolonged in patients. VAT was found to have the strongest association with thrombin generation parameters: lag time (β = 0.378; *p* < 0.001), peak thrombin (0.378; *p* = 0.04), and ttP (β = 0.373; *p* = 0.001). BMI was found to be a predictor for lag time only (β = 0.313; *p* = 0.003). SAT was not associated with any of the thrombin generation parameters (data not shown). VAT was found to be an independent determinant of peak thrombin, lag time, and ttP. The study suggests not only fat mass but also fat distribution, particularly visceral adiposity, mediates hypercoagulability in obesity.

## Introduction and Background

Obesity is recognized as a growing global concern with a worldwide prevalence for overweight and obesity approaching 40% in adults. 1 Morbid obesity (body mass index [BMI] >40 kg/m ^2^ ) has been described in 3.9% of women and 1.6% of men in the United Kingdom. 2 There is a significantly increased risk of cardiovascular disease, type 2 diabetes, hypertension, and venous thromboembolism in obesity, particularly when severe. 3

Several mechanisms have been proposed to explain the increased risk of venous and arterial thromboembolism, including increased procoagulant activity (factor [F] VII, FVIII, von Willebrand factor, and fibrinogen), impaired fibrinolysis (increased plasminogen activator inhibitor [PAI]), increased inflammation, endothelial dysfunction, and altered lipid and glucose metabolism in metabolic syndrome. 4 Inflammation and insulin resistance are at least in part mediated by adipocytokines, 4 5 with those released by visceral rather than subcutaneous tissue directly associated with metabolic abnormalities. 6

Increased visceral adipose tissue (VAT) has been shown to be associated with the development of insulin resistance, type 2 diabetes, stroke, and ischemic heart disease. 7 8 9 BMI and other conventional anthropometric measures, such as waist circumference, are insensitive to fat distribution pattern 10 11 and do not reflect recognized age, gender, and ethnic-specific differences in fat distribution. 12 13 14 It remains unknown whether fat distribution impacts on coagulation markers and/or the risk of venous thrombosis.

Adipose tissue is known to produce several cytokines including leptin and adiponectin, tumor necrosis factor-α, and PAI-1. 15 Leptin is predominantly but not specifically produced and secreted by adipose tissue. 4 Adiponectin, in contrast, is exclusively produced by adipose tissue. Adiponectin levels correlate negatively with obesity and recent work has shown adiponectin release to be reduced from the VAT of obese subjects (with no change in release from subcutaneous fat). 6 16 There are no studies examining the relationship of these adipocytokines and risk of VTE in morbidly obese patients.

Global coagulation assays, such as thrombin generation, can provide an indirect measure of overall “coagulability” and have been demonstrated to be sensitive to the prothrombotic state associated with increased age, hormone use, and pregnancy. Recent studies have reported increased thrombin generation in morbid obesity with a reduction following weight loss, suggesting adiposity mediates the hypercoagulable state. 17 18 This study aims to evaluate markers of hypercoagulability in grade III obesity (BMI >40 kg/m ^2^ ) compared with nonobese controls. We further sought to determine whether hypercoagulability was influenced by VAT, metabolic syndrome, and metabolic markers, including adiponectin.

## Patients and Methods

### Study Participants

Ninety patients were recruited from the obesity clinic at King's College Hospital NHS Foundation Trust from November 2009 to December 2011. The inclusion criteria were class III obesity (BMI ≥40 kg/m ^2^ ) and age 18 to 65 years. A control group (healthy ambulatory participants, with a BMI < 30 kg/m ^2^ ) was recruited from volunteers responding to advertisement. The control participants were matched to cases for age and sex. Exclusion criteria for all participants were personal history of VTE, known active cancer, previous history of myocardial infarction, cerebrovascular accident or peripheral vascular disease, current use of hormone replacement therapy (HRT), oral contraceptives or anticoagulants, previous bariatric surgery, and current pregnancy.

Body weight and height were measured to enable calculation of BMI. Blood pressure (BP) was recorded using the OxiMax Nellcor spot signs device (Nellcor OxiMax, New York, United States); this was measured in the right arm with the subject in a sitting position. Participants had their BP measured after a 15-minute rest period. Data regarding participant's self-reported ethnicity, medical history, and smoking status were also collected. Fasting blood samples were collected from all participants at their convenience following a ≥ 10-hour fasting period. Visceral adiposity was evaluated by computed tomography scan as detailed below.

The study was approved by King's College Research Ethics Committee (REC: 09/H0808/64) and the Research and Development Committee. All participants gave written informed consent prior to participation.

### Blood Collection

Venous blood was drawn from an antecubital vein using a plastic syringe connected to a 21 g butterfly system (Hospira Venisystems, Illinois, United States). Blood was immediately dispensed into 0.109 mol/L sodium citrate tubes (Vacutainer BD, Plymouth, UK) following an initial 10 mL blood drawn, which was subsequently dispensed into 0.369 EDTA (Vacutainer BD, Plymouth), SST II (Vacutainer BD, Plymouth), and sodium fluoride tube (Vacutainer BD, Plymouth).

### Sample Preparation

Platelet poor plasma (PPP) for standard coagulation assays was prepared within an hour of sample collection by double centrifugation (7 minutes) at 1,700 *g* (Hettich 38R Rotina centrifuge; Hettich-Zentrifugen, Tuttlingen, Germany). Prothrombin time (PT), activated partial thromboplastin time (APTT), Clauss fibrinogen (CFIB), and D-dimer (DD) were measured in fresh unfrozen plasma within 4 hours of sample collection. Aliquots (2 mL) were immediately frozen at −40°C and only thawed by incubating in a water bath at 37°C immediately before testing. Metabolic markers were measured using serum separated by centrifugation of fresh blood collected in SST II tubes for 7 minutes at 1,700 *g* (38R Rotina centrifuge [Hettich-Zentrifugen]). All patient and control samples were assayed by the same experienced biomedical scientists.

PPP for thrombin generation was prepared by double centrifugation at 4,754 *g* for 10 minutes (420R Rotina centrifuge (Hettich-Zentrifugen). After the first spin, the top three-quarter volume of supernatant was transferred into a polypropylene tube before second spin. PPP was then frozen at −40°C within 45 minutes of sample collection. Samples were thawed at 37°C immediately before analysis. All samples were analyzed within 2 months of collection.

### Laboratory Measurements

#### Metabolic Analysis

Total cholesterol, triglycerides, and high-density lipoprotein (HDL) were determined using Siemens kits (Chol-c, Trig and D-HDL kits [Siemens Diagnostics, Illinois, United States]), respectively, on the ADVIA2400 (Siemens Diagnostics). Manufacturer protocols were employed in all testing.

Fasting insulin was measured using a solid phase chemiluminescent assay on the Siemens Immulite 2000 analyzer (Siemens Healthcare Diagnostics). The original homeostasis model assessment index (HOMA-IR: HOMA-IR = [fasting glucose (mmol/L) × fasting insulin (mIU/L)]/22.5) was used to estimate insulin resistance among the patients. Patients with HOMA-IR > 2.4 were classified as having insulin resistance. 19

Metabolic syndrome was defined using the International Diabetes Federation (IDF) guidelines: 20 presence of central obesity (assumed for patients with BMI ≥ 30 kg/m ^2^ ) plus any two of the following four conditions—hypertriglyceridemia, ≥ 1.7 mmol/L; low HDL cholesterol, < 1.03 mmol/L (men) and < 1.29 (women); high BP, ≥ 130 mm Hg systolic pressure or ≥ 85 mm Hg diastolic pressure; or fasting glucose, > 5.6 mmol/L. Previously diagnosed or patients on treatment for hypertension, diabetes, or hyperlipidemia were considered to satisfy the criterion.

Serum levels of adiponectin were measured using an ELISA kit (total human Adiponectin/Acrp 30 Quantikine ELISA; R&D Systems, Minneapolis, Minnesota, United States).

### Coagulation Tests

PT, APTT, and CFIB were measured using STA Neoplastine C1 plus, STA Cephascreen, and STA fibrinogen in clot-based assays, respectively. Antithrombin (AT), protein C (PC), free protein S (FPS), and DD were assayed using STA-Stachrom ATIII, STA-Stachrom protein C, Liatest Free Protein S, and STA Liatest D-Di. Colorimetric technique was employed for AT and PC, while latex immunoassay technology was employed for DD and FPS. FVII and FVIII were assayed using STA deficient VII/VIII immunodepleted plasma in coagulation-based assays. All reagents were purchased from Diagnostica Stago (Asniers, France) with assays performed on STA-R Evolution analyzer (Diagnostica Stago) according to manufacturer protocols and as previously described. 21

Free tissue factor pathway inhibitor (TFPI) was measured using an ELISA (Asserachrom Free TFPI, Diagnostica Stago). PAI was also measured with ELISA (Human Serpin E1/PAI-1 Quantikine ELISA kit (DSE100), R&D Systems).

### Thrombin Generation Assay

Thrombin generation was measured using the calibrated automated thrombography method as described in detail by Hemker et al. 21 22 PPP reagent (5 pM; Thrombinoscope BV, Maastricht, the Netherlands) and the thrombin calibrator were reconstituted in 1 mL of distilled water. The calibrator is made of a thrombin-like substance that is able to cleave the fluorescent substrate (Z-Gly-Gly-Arg-AMC; Bachem, Bubendorf, Switzerland). Eighty microliters of PPP, prewarmed to 37°C, was added to 20 µL of either PPP reagent (5pM; Thrombinoscope BV) or calibrator (Thrombin Calibrator, Thrombinoscope BV) at room temperature in a 96-well microtiter plate (Immulon 2HB; Thermo Electron Corporation, Vantaa, Finland) to give a final concentration of 5 pm tissue factor and 4 µM phospholipids. Both calibrator and test wells were processed in triplicate. Thrombin generation was then initiated by adding 20 µL of the fluorogenic substrate (FluCa, Thrombinoscope BV) containing 100 mM calcium chloride. A Fluoroskan Ascent plate reader (Thermo Labsystems, Helsinki, Finland); excitation filter of 390 nm and emission filter of 460 nm, and Thrombinoscope software (Thrombinoscope BV) were used for the assay following manufacturer's instructions. The continuous production of thrombin was determined by measuring fluorescence every 20 seconds for a period of 1 hour. The assay yields four thrombin generation parameters: lag time, ttP, peak thrombin, and endogenous thrombin potential (ETP; area under the curve, nM/min) (Thrombinoscope software version 3.1.0.55; Synapse BV, Maastricht, the Netherlands). Furthermore, Velocity Index = Peak/(ttP − Lag Time), indicating the average net rate of prothrombin activation during the propagation phase, was calculated. Control plasma (Normal Human Control Plasma, Technoclone, Vienna, Austria) was utilized in each thrombin generation assay and this was utilized to evaluate interassay variability across all tests. Intra-assay variability was <5% and interassay variability was <10% for all parameters.

### Fat Distribution Analysis

#### Computed Tomographic Scans

A Somatom Sensation 16 CT scanner (Siemens, Forchheim, Germany) was used to take a noncontrast single-slice CT scan at the midpoint of the L4 vertebral body. The patients were examined in supine position with their arms extended above their heads. The CT protocol used was as follows: slice thickness, 10 mm; scan parameters, 120 kv, 120 mA; field of view, 499 mm with a 512 × 512 matrix; window level, 300; center, 40. The images were transferred onto CDs in DICOM (Digital Imaging and Communications in Medicine) format to allow for analysis.

#### Image Analysis

**Fig. 1:**
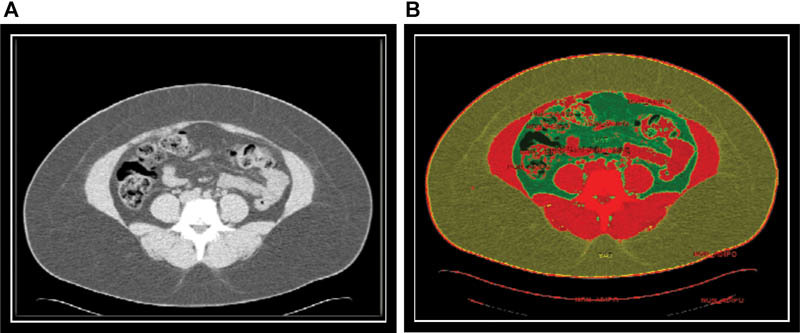
Measurement of visceral and subcutaneous adipose tissue. ( **A** ) Screen capture from the region of interest module before segmenting. ( **B** ) Screen capture after segmenting VAT (green area) and SAT (yellow area).

Semiautomated assessment of VAT and SAT compartments was performed using a dedicated software package, Analyze direct 10 (AnalyzeDirect, Kansas, United States). The measurement of adipose tissue area was determined by setting a Hounsfield unit (HU) range between −190 and −30. 23 SAT was defined as fat that is superficial to abdominal wall musculature, whereas VAT was defined as adipose tissue inside the muscular wall and included the mesenteric, subperitoneal, and intraperitoneal components. All the images were analyzed by only one observer and the intraobserver variability for one image examined 10 times on the same day was < 5% for VAT areas ( Fig. 1 ).

### Statistical Analysis

Normality on continuous variables was evaluated with the Shapiro–Wilk test and normality Q-Q plots. Continuous variables are given as mean and standard deviation or median and interquartile ranges for normal and nonnormal variables, respectively. Nonnormal variables were logarithmically transformed to normalize where possible. The means of continuous variables were compared using the independent Student's *t* -test. Fisher's exact test was used to assess the differences between nominal variables for potential confounders on thrombin generation. Correlations between continuous variables were evaluated with Pearson's correlation for normal data and Spearman's correlation for nonnormal data. Simple and multiple linear regression analyses were performed to assess the association of obesity markers, metabolic markers, and thrombin generation parameters within the morbidly obese group. The dependent variable was always the thrombin generation parameter. Statistical significance was given a *p* -value of < 0.05. All statistical analyses were performed using SPSS version 19 software (IBM SPSS, Chicago, Illinois, United States).

## Results

### Study Population

Ninety subjects were recruited, of which one did not attend for blood tests and was thus excluded, along with 77 age- and sex-matched controls. Baseline anthropometric parameters of morbidly obese cases and controls are shown in Table 1 . There was no significant difference in age, gender, and smoking status between the two groups. Subjects had higher mean systolic and diastolic blood pressure as shown in Table 1 . The mean fasting insulin, mean glucose, median triglycerides, and median HOMA-IR were significantly increased in subjects with significantly depressed mean levels of HDL and adiponectin. Twenty-one (24%) subjects were taking antihypertensive medication 16 (18%) hypoglycemic agents, and 6 (7%) statin therapy. Fifty-three (60%) subjects met the criteria for metabolic syndrome.

**Table 1:** Anthropometric and metabolic parameters for the study population

Parameter	Cases ( *N* = 89)	Controls ( *N* = 77)
Mean age, y (SD)	44 ± 11	42 ± 11
Male sex, *n* (%)	15 (17)	19 (25)
Ethnicity		
Caucasian	53 (59.6)	47 (61)
African Caribbean	32 (36)	19 (24.7)
Other	4 (4.5)	11 (14.3)
Smokers, *n* (%)	15 (17)	10 (13)
Mean systolic blood pressure (mm Hg)	130 ± 13	115 ± 12**
Mean diastolic blood pressure (mm Hg)	79 ± 8	73 ± 9**
Mean BMI (kg/m ^2^ )	50.5 ± 6.9	24 ± 3**
Mean VAT (cm ^2^ )	242.75 ± 89.65	71.27 ± 28.15**
Mean SAT (cm ^2^ ) a	710.97 ± 119.87	188.47 ± 88.55**
Mean height (cm)	166 ± 8	168 ± 9
Thrombophilia, *n* (%)		
Present	8 (9)	7 (9.1)
FVL	4 (4.5)	1 (1.3)
PT G20210A	1 (1.1)	4 (5.2)
Lupus anticoagulant	3 (3.4)	2 (2.6)
Median glucose (mmol/L)	5.5 (4.9–6.6)	4.8 (4.6–5.1)**
Mean fasting insulin (µU/mL)	18.8 ± 10.4	3.9 ± 0.7**
Median HOMA IR	4.6 (3.06–7.11)	0.51 (0.41–1.07)**
Median triglycerides (mmol/L)	1.3 (0.9–1.8)	0.8 (0.6–0.9)**
Mean HDL (mmol/L)	1.2 ± 0.3	1.7 ± 0.4**
Mean LDL (mmol/L)	2.9 ± 0.9	2.9 ± 0.8
Mean total cholesterol (mmol/L)	4.8 ± 1.0	5.0 ± 0.9
Median adiponectin (ng/mL)	5,366 (3,880–7,635)	9,461(4,844–14,400)**

Abbreviations: BMI, body mass index; FVL, factor V Leiden heterozygote; HDL, high-density lipoproteins; HOMA-IR, homeostasis model assessment index for insulin resistance; LDL, low-density lipoproteins; N, population; PT, prothrombin G20210A heterozygote; SAT, subcutaneous adipose tissue area; SD, standard deviation; VAT, visceral adipose tissue area; VTE, venous thromboembolism.

Notes: Continuous normally distributed data are presented as mean ± SD; nonnormally distributed data are presented as median (interquartile range). *p* -Values are based on unpaired Student *t* -test for normally distributed continuous variables, Mann–Whitney for nonnormally distributed continuous variables, and Fisher's exact test for nominal variables. Significant differences are indicated as follows: ** *p* < 0.001; *0.05 < *p* ≥ 0.001.

a SAT measured in 69 cases only due to inability for 20 subjects to fit into CT scanner.

### Hemostatic Markers

There were significant differences in the activity of coagulation factors and natural anticoagulant as shown in Table 2 , with FVII, FVIII, PC, FPS, and TFPI significantly increased in morbid obesity and AT significantly reduced. Markers of fibrinolysis, PAI, and DD were found to be significantly enhanced in morbid obesity as shown in Table 2 .

**Table 2:** General Hemostatic markers for the study population

Variable	Cases ( *N* = 89)	Control ( *N* = 77)
Mean Fib (mg/L)	4.2 ± 0.8	3.1 ± 0.7**
Mean FVII (U/dL)	129 ± 29	104 ± 23**
Mean AT (%)	96 ± 10	101 ± 8*
Mean PC (U/dL)	116 ± 19	106 ± 20*
Mean FPS (%)	104 ± 24	91 ± 23*
Median FVIII (IU/dL)	148 (126–195)	121 (98–144)**
Median DD (mg/L)	420 (315–670)	280 (220–413)**
Median PAI-1 (ng/mL)	5.51 (3.19–9.11)	1.54 (0.83–2.71)**
Mean TFPI (ng/mL)	14.1 ± 4.91	10.37 ± 4.51**

Abbreviations: AT, antithrombin activity; DD, D-dimer; Fib, Clauss fibrinogen; FPS, free protein S; N, number; PAI-1, plasminogen activator inhibitor 1; PC, protein C activity; PLT, platelet count; TFPI, tissue factor pathway inhibitor.

Notes: Normally distributed data are presented as mean ± SD (standard deviation) and the *p* -values are based on independent Student's *t* -test. Nonnormally distributed data are presented as median (interquartile range) and *p* -values are based on test. Significant differences are indicated as follows: ** *p* < 0.001; *0.05 < *p* ≥ 0.001.

**Fig. 2:**
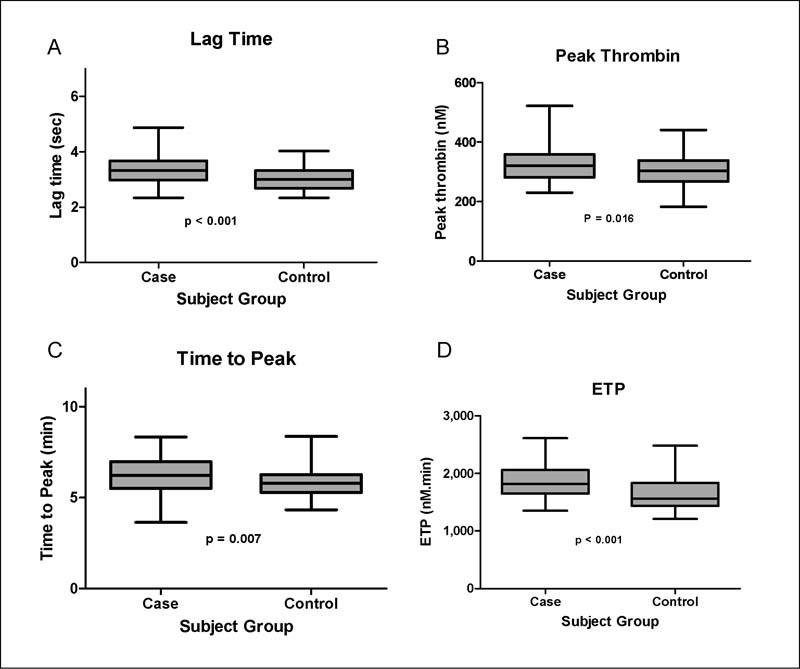
Comparison of thrombin generation parameters in cases and controls. ( **A** ) Mean lag time in cases was significantly higher than in the control group: 3.33 ± 0.56 versus 3.0 ± 0.41, *p* < 0.001. ( **B** ) Mean peak thrombin was higher in the cases compared with the control group: 322 ± 54 versus 304 ± 55, *p* = 0.016. ( **C** ) Mean time to peak was higher in cases than in the control group: 6.20 ± 0.99 versus 5.77 ± 0.81, *p* = 0.007. ( **D** ) Cases had a significantly higher mean ETP compared with the control group: 1,854 ± 287 versus 1660 ± 282, *p* < 0.001.

Thrombin generation revealed a significantly increased peak and ETP in subjects compared with controls as shown in Fig. 2 . Lag time was significantly prolonged in subjects ( Fig. 2 ).

#### Fat Distribution Markers as Determinants of Hypercoagulability Markers in Morbid Obesity

VAT was significantly increased in the morbidly obese ( Table 1 ). Within the obese cohort, there were positive correlations between VAT, FVIII, fibrinogen, free protein S, and TFPI and a negative correlation with AT ( Table 3 ). VAT also positively correlated with thrombin generation, lag time, and peak thrombin as shown in Table 4 . BMI was significantly correlated with FVIII, fibrinogen, TFPI, DD, and lag time. There were no correlations between SAT and any of the hemostatic markers (data not shown). There was no significant difference in the velocity index between the patient group and control group (data not shown). Within the patient group, velocity index was positively correlated with FVIII (0.219, *p* = 0.04) and negatively with FVII (−0.213, *p* = 0.04).

**Table 3:** Correlation between fat distribution markers and general hemostatic markers in the morbidly obese

Variables	F VII	F VIII _log_	FIB	AT	PC	PAI _log_	TFPI	FPS	D-dimer _log_
BMI	0.017	0.281*	0.323*	−0.131	−0.047	−0.071	0.246*	0.096	0.202*
VAT	0.111	0.253*	0.263*	−0.275*	−0.009	0.043	0.341**	0.337*	−0.003

Abbreviations: AT, antithrombin; BMI, body mass index; FIB, fibrinogen; FPS, free protein S; FVII, factor VII; FVIII, factor VIII; PAI, plasminogen activator inhibitor; PC, protein C activity; TFPI, tissue factor pathway inhibitor; VAT, visceral adipose tissue area.

Notes: Data are Pearson's correlation coefficients (R). Variables (FVIII, D-dimer, and PAI) were not normally distributed and logarithmic transformation (log) was performed. Significant coefficients are indicated as follows: ** *p* < 0.001; *0.05 < *p* ≥ 0.001.

**Table 4:** Correlation between fat distribution markers and thrombin generation parameters in morbid obesity

Variable	Lag time	ETP	Peak	ttP
BMI	0.313*	0.097	−0.058	0.127
VAT	0.411**	−0.039	0.257*	0.360*

Abbreviations: BMI, body mass index; ETP, endogenous thrombin potential; ttP, time to peak; VAT, visceral adipose tissue area.

Notes: Data are Pearson's correlations (R). Significant correlation coefficients are indicated as follows: ** *p* < 0.001; *0.05 < *p* ≥ 0.001.

Multiple stepwise regression analysis (adjusted for age, smoking status, gender, and ethnicity) was used to assess the strength of the relationships between the fat distribution markers and thrombin generation parameters. VAT was found to have the strongest association with thrombin generation parameters: lag time (β = 0.378; *p* < 0.001), peak thrombin (0.378; *p* = 0.04), and (β = 0.373; *p* = 0.001). BMI was found to be a predictor for lag time only (β = 0.313; *p* = 0.003). SAT was not associated with any of the thrombin generation parameters (data not shown).

#### Metabolic Syndrome, Its Components, and Hypercoagulability Markers

Of the 89 participants, 50 (56%) had metabolic syndrome. There were no significant differences in gender, ethnicity, BMI, or smoking status between those with and without metabolic syndrome (data not shown). Individuals with the metabolic syndrome were older (47 ± 11 vs. 42 ± 11 years, *p* = 0.038) and had higher VAT (265 ± 95 cm ^2^ vs. 215 ± 75 cm ^2^ , *p* = 0.01) compared with those without metabolic syndrome. On comparing hemostatic markers, TFPI and PC were the only hemostatic markers that were significantly raised in those with metabolic syndrome compared to those without (12.7 ± 4.1 vs. 15.2 ± 5.3, *p* = 0.012 and 111 ± 18 vs. 119 ± 20, *p* =  0.05, respectively). There were no differences in thrombin generation parameters seen (data not shown). There was a positive correlation between glucose and ETP ( *r* = 0.259, *p* = 0.003), and between triglycerides and ttP ( *r* = 0.230, *p* = 0.031). There were no correlations seen between thrombin generation parameters and other metabolic markers (insulin, HOMA-IR, HDL) or adiponectin (data not shown). There was no significant difference in adiponectin between those with and without metabolic syndrome and no correlation with VAT (data not shown). VAT remained a significant independent determinant of peak thrombin, lag time, and after adjusting for metabolic markers as shown in Table 5 .

**Table 5:** Multiple regression models of anthropometric, biochemical markers as determinants of thrombin generation

	ETP	Lag time	ttP	Peak
VAT	ns	0.341*	0.212*	0.204*
Systolic BP	0.266*	ns	ns	0.247*
Diastolic BP	ns	ns	ns	0.236*
Glucose	0.259*	ns	ns	ns
Triglycerides	ns	ns	0.244*	ns
HDL	ns	ns	ns	ns
Cholesterol	0.264*	0.234*	0.275*	ns

Abbreviations: BP, blood pressure; ETP, endogenous thrombin potential; HDL, high-density lipoprotein; ns, nonsignificant; ttP, time to peak; VAT, visceral adipose tissue area.

Notes: For regression multivariate model, age, gender, ethnicity, and smoking status were included as independent variables. Significant results are indicated as follows: ** *p* < 0.001; *0.05 < *p* ≥ 0.001.

## Discussion

We have demonstrated an altered thrombin generation profile in morbid obesity, with prolonged lag time, time to peak, and increased peak thrombin and ETP. Additionally, FVII, FVIII, fibrinogen, DD, PC, FPS, PAI, and TFPI were increased in the morbidly obese with reduced AT compared with nonobese individuals in keeping with previous reports. 24 25 Our findings confirm those reported by two other groups during the course of our study, which demonstrated increased thrombin generation in the morbidly obese. 17 18 Weight loss following bariatric surgery was additionally associated with a significant reduction in the hypercoagulable markers of thrombin generation. 17

We observed an unexpected prolongation of thrombin generation, lag time, and time to peak in the morbidly obese, in contrast to the findings of Campello and colleagues, 17 who reported a shortened lag time. Of note, Ay et al 16 did not compare with a control group and lag times reported in the subject group prior to bariatric surgery were increased compared with those seen in our study (this may be, in part, due to different thrombin generation methodology). This group reported a reduction in lag time following weight loss. 17 Fibrinogen, FVII, FPS, and free TFPI have previously been reported as independent determinants of lag time in healthy subjects measured at high tissue factor concentrations. 26 We observed significantly increased levels of these parameters in the morbidly obese group compared with controls, with positive correlations between these parameters and VAT. We propose that the increase in these parameters is responsible for an in vitro effect of prolonged lag time. This is supported by the findings of Brodin and colleagues 26 that increasing concentrations of TFPI increase lag time with no effect on ETP or peak thrombin. The same group reported that removal of native TFPI shortens the lag time with no change on ETP and peak thrombin. TFPI acts on activated FX (a) and VIIa-TF-Xa to regulate the initiation phase of thrombin generation. FPS inhibits thrombin generation independent of activated protein C by potentiating TFPI's activity. 28 Increased DDs have also been reported to have an in vitro effect of prolonging lag time in patients with acute myocardial infarction. 29 DDs were higher in our morbidly obese group and this may be additional contributor to the prolonged lag time observed. Of note, this is an in vitro effect; increased DD, FPS, and TFPI are not associated with bleeding in healthy individuals. Ttp is determined by initiation of coagulation (lag time) and rate of thrombin generation (velocity index); as there was no difference in velocity index between morbidly obese and controls, the prolonged ttP is a consequence of the initial prolongation in lag time.

Our study additionally investigated how morbid obesity and fat distribution patterns influence known markers of hypercoagulability. We observed positive correlations between FVII, FVIII, fibrinogen, and FPS; both BMI and VAT; and a negative correlation with antithrombin, in keeping with previous reports. 26 Correlations were slightly stronger with BMI compared with VAT. Given both procoagulant factors and natural anticoagulants were increased in the morbidly obese cohort, it is difficult to evaluate how each of these individual factors influence overall thrombotic risk. This is overcome by measuring thrombin generation which provides an overall view of “coagulability” as described earlier. 30 VAT was found to be an independent determinant of peak thrombin, lag time, and ttP. Although prolongation of the lag time might suggest hypocoagulability, previous studies evaluating thrombotic risk suggest ETP and peak thrombin provide the most consistent results and are associated with both first and recurrent VTE. 22 31 32 33 34 35 This suggests that fat distribution and, specifically, VAT may therefore modulate the risk of venous thrombosis, similar to previous reports of increased cardiovascular morbidity and mortality associated with increased VAT. 7 8 9

VAT is known to have endocrine function and to mediate adipocytokine expression associated with metabolic abnormalities in obesity. 36 We measured adiponectin, which is inversely related to body fat mass and insulin resistance. Low circulating levels are strongly associated with metabolic syndrome. 5 Adiponectin has been shown to directly modulate endothelial function by reducing the inflammatory response to vascular injury with low levels reported as associated with increased risk of coronary artery disease. 37 Adiponectin was significantly lower in the morbidly obese patients compared with controls. However, there was no significant difference between those with or without metabolic syndrome and adiponectin levels did not correlate with VAT in our study. It is perhaps unsurprising that we did not therefore observe any correlation between adiponectin and thrombin generation parameters.

We did not identify any direct association between the presence of metabolic syndrome and thrombin generation parameters. This supports findings of Campello et al, 17 who found no association between metabolic syndrome and thrombin generation but a close relationship with BMI and waist circumference (but not other components of the metabolic syndrome) leading them to conclude that hypercoagulability is closely related to fat mass. 18 In contrast, we found no relationship between BMI and metabolic syndrome, but identified significantly increased VAT in those with metabolic syndrome. Our study suggests not only fat mass but also fat distribution mediates hypercoagulability in obesity. Of note, this finding may have been influenced by the unselected inclusion of participants with morbid obesity; while none had undergone bariatric surgery, many were already on treatment for comorbidities associated with obesity such as hypertension, diabetes, or hyperlipidemia. However, upon examining individual components of the metabolic syndrome, we observed the hypertension criterion as an independent determinant of both peak thrombin and ETP, with fasting glucose also significantly contributing to ETP, and triglycerides to ttP. There is increasing evidence to suggest hyperglycemia constitutes a prothrombotic state. 37 To our knowledge, thrombin generation has not been specifically studied in hypertension, hypertriglyceridemia, or hyperglycemia.

### Limitations

Due to the cohort size, and as we did not measure all coagulation factors, we were unable to evaluate independent determinants of thrombin generation parameters in the morbidly obese. However, our aim was to establish whether morbid obesity is associated with hypercoagulability and the role of individual coagulation markers in evaluating this is dubious. While there was no significant difference in age or sex between groups, there was a greater proportion of males in the control group. Healthy females have lower FPS concentrations compared with men. 26 However, as FPS was lower in the control group, which had the smaller proportion of females, this does not explain the difference detected between groups.

We used a high concentration, 5 pM of tissue factor for thrombin generation and did not use thrombomodulin/Protac to evaluate the protein C pathway. At the time of study, lower concentrations of tissue factor (≤1 pM) were thought to be associated with contact activation and collection of samples with corn trypsin inhibitor (CTI) was considered necessary. We selected a higher concentration of tissue factor to avoid the need for CTI and potentially improve the reproducibility of the study. Similarly, the use of Protac/thrombomodulin was not standardized and we did not evaluate TG in its presence, we are therefore not able to comment on the significance of the protein C pathway. The additional use of these thrombin generation assays may have enabled further elucidation of the effects of increased FPS and TFPI observed in the morbidly obese. We did not measure other adipocytokines or inflammatory markers and are therefore unable to comment further on the mechanism by which VAT mediates hypercoagulability. Adiponectin, the cytokine produced most specifically by VAT, was not associated with thrombin generation parameters. Other studies have reported positive correlations between tumor necrosis factor-α, leptin, CRP and ETP. 18

## Conclusion

Morbid obesity is associated with an altered thrombin generation profile suggestive of hypercoagulability with increased peak thrombin. Hypercoagulability appears to be mediated by visceral adiposity, with VAT being a strong determinant for peak thrombin. We suggest this relates to endocrine function of VAT; further research is required to establish the underlying mechanism.

## References

[1] Health and Social Care Information Centre.The Health Survey for England - 2012 Trend TablesLondon: 2013. Available at:http://www.hscic.gov.uk/catalogue/PUB13219. Accessed May 15, 2017

[2] Stein P D Beemath A Olson R E Obesity as a risk factor in venous thromboembolism Am J Med 2005 118 09 978 980 16164883 10.1016/j.amjmed.2005.03.012

[3] Darvall K A Sam R C Silverman S H Bradbury A W Adam D J Obesity and thrombosis Eur J Vasc Endovasc Surg 2007 33 02 223 233 17185009 10.1016/j.ejvs.2006.10.006

[4] Fisman E Z Tenenbaum A Adiponectin: a manifold therapeutic target for metabolic syndrome, diabetes, and coronary disease? Cardiovasc Diabetol 2014 13 103 24957699 10.1186/1475-2840-13-103 PMC4230016

[5] Drolet R Bélanger C Fortier M Fat depot-specific impact of visceral obesity on adipocyte adiponectin release in women Obesity (Silver Spring) 2009 17 03 424 430 19219061 10.1038/oby.2008.555

[6] Karcher H S Holzwarth R Mueller H P Body fat distribution as a risk factor for cerebrovascular disease: an MRI-based body fat quantification study Cerebrovasc Dis 2013 35 04 341 348 23615579 10.1159/000348703

[7] Yang L Samarasinghe Y P Kane P Amiel S A Aylwin S J Visceral adiposity is closely correlated with neck circumference and represents a significant indicator of insulin resistance in WHO grade III obesity Clin Endocrinol (Oxf) 2010 73 02 197 200 20050862 10.1111/j.1365-2265.2009.03772.x

[8] Brochu M Starling R D Tchernof A VAT is additionally an independent predictor of all-cause mortality in men Obesity (Silver Spring) 2000 14 336 341

[9] van der Kooy K Seidell J C Techniques for the measurement of visceral fat: a practical guide Int J Obes Relat Metab Disord 1993 17 04 187 196 8387967

[10] Sowers J R Obesity as a cardiovascular risk factor Am J Med 2003 115(115, Suppl 8A):37S 41S 14678864 10.1016/j.amjmed.2003.08.012

[11] Lovejoy J C de la Bretonne J A Klemperer M Tulley R Abdominal fat distribution and metabolic risk factors: effects of race Metabolism 1996 45 09 1119 1124 8781299 10.1016/s0026-0495(96)90011-6

[12] Gallagher D Visser M Sepúlveda D Pierson R N Harris T Heymsfield S B How useful is body mass index for comparison of body fatness across age, sex, and ethnic groups? Am J Epidemiol 1996 143 03 228 239 8561156 10.1093/oxfordjournals.aje.a008733

[13] Rush E C Goedecke J H Jennings C BMI, fat and muscle differences in urban women of five ethnicities from two countries Int J Obes 2007 31 08 1232 1239 10.1038/sj.ijo.0803576 17342075

[14] Ottaviani E Malagoli D Franceschi C The evolution of the adipose tissue: a neglected enigma Gen Comp Endocrinol 2011 174 01 1 4 21781968 10.1016/j.ygcen.2011.06.018

[15] Motoshimo H Wu X Sinha M K Differential regulation of adiponectin secretion from cultured human omental and subcutaneous adipocytes: effects of insulin and rosaglitazone J Clin Endocrinol Metab 2002 87 12 5662 5667 12466369 10.1210/jc.2002-020635

[16] Ay L Kopp H P Brix J M Thrombin generation in morbid obesity: significant reduction after weight loss J Thromb Haemost 2010 8 04 759 765 20102484 10.1111/j.1538-7836.2010.03766.x

[17] Campello E Zabeo E Radu C M Hypercoagulability in overweight and obese subjects who are asymptomatic for thrombotic events Thromb Haemost 2015 113 01 85 96 25318550 10.1160/TH14-02-0156

[18] Matthews D R Hosker J P Rudenski A S Naylor B A Treacher D F Turner R C Homeostasis model assessment: insulin resistance and beta-cell function from fasting plasma glucose and insulin concentrations in man Diabetologia 1985 28 07 412 419 3899825 10.1007/BF00280883

[19] International Diabetes Federation.The IDF Consensus Worldwide Definition of the Metabolic SyndromeAvailable at:http://www.idf.org/webdata/docs/MetS_def_update2006.pdf. Accessed May 15, 2017

[20] Bagot C N Marsh M S Whitehead M The effect of estrone on thrombin generation may explain the different thrombotic risk between oral and transdermal hormone replacement therapy J Thromb Haemost 2010 8 08 1736 1744 20553380 10.1111/j.1538-7836.2010.03953.x

[21] Hemker H C Giesen P AlDieri R The calibrated automated thrombogram (CAT): a universal routine test for hyper- and hypocoagulability Pathophysiol Haemost Thromb 2002 32(5-6):249 253 13679651 10.1159/000073575

[22] Kuk J L Church T S Blair S N Ross R Does measurement site for visceral and abdominal subcutaneous adipose tissue alter associations with the metabolic syndrome? Diabetes Care 2006 29 03 679 684 16505526 10.2337/diacare.29.03.06.dc05-1500

[23] Kaye S M Pietiläinen K H Kotronen A Obesity-related derangements of coagulation and fibrinolysis: a study of obesity-discordant monozygotic twin pairs Obesity (Silver Spring) 2012 20 01 88 94 21959347 10.1038/oby.2011.287

[24] Cugno M Castelli R Mari D Inflammatory and prothrombotic parameters in normotensive non-diabetic obese women: effect of weight loss obtained by gastric banding Intern Emerg Med 2012 7 03 237 242 21249470 10.1007/s11739-011-0522-x

[25] Dielis A W Castoldi E Spronk H M Coagulation factors and the protein C system as determinants of thrombin generation in a normal population J Thromb Haemost 2008 6 01 125 131 17988231 10.1111/j.1538-7836.2007.02824.x

[26] Brodin E Appelbom H Osterud B Hilden I Petersen L C Hansen J B Regulation of thrombin generation by TFPI in plasma without and with heparin Transl Res 2009 153 03 124 131 19218095 10.1016/j.trsl.2008.12.004

[27] Hackeng T M Seré K M Tans G Rosing J Protein S stimulates inhibition of the tissue factor pathway by tissue factor pathway inhibitor Proc Natl Acad Sci U S A 2006 103 09 3106 3111 16488980 10.1073/pnas.0504240103 PMC1413864

[28] Smid M Dielis A W Winkens M Thrombin generation in patients with a first acute myocardial infarction J Thromb Haemost 2011 9 03 450 456 21143375 10.1111/j.1538-7836.2010.04162.x

[29] van Veen J J Gatt A Makris M Thrombin generation testing in routine clinical practice: are we there yet? Br J Haematol 2008 142 06 889 903 18564356 10.1111/j.1365-2141.2008.07267.x

[30] van Hylckama Vlieg A Christiansen S C Luddington R Cannegieter S C Rosendaal F R Baglin T P Elevated endogenous thrombin potential is associated with an increased risk of a first deep venous thrombosis but not with the risk of recurrence Br J Haematol 2007 138 06 769 774 17760809 10.1111/j.1365-2141.2007.06738.x

[31] Hron G Kollars M Binder B R Eichinger S Kyrle P A Identification of patients at low risk for recurrent venous thromboembolism by measuring thrombin generation JAMA 2006 296 04 397 402 16868297 10.1001/jama.296.4.397

[32] Tripodi A Legnani C Chantarangkul V Cosmi B Palareti G Mannucci P M High thrombin generation measured in the presence of thrombomodulin is associated with an increased risk of recurrent venous thromboembolism J Thromb Haemost 2008 6 08 1327 1333 18485081 10.1111/j.1538-7836.2008.03018.x

[33] Tripodi A Legnani C Palareti G Chantarangkul V Mannucci P M More on: high thrombin generation and the risk of recurrent venous thromboembolism J Thromb Haemost 2009 7 05 906 907 19320819 10.1111/j.1538-7836.2009.03338.x

[34] Dargaud Y Trzeciak M C Bordet J C Ninet J Negrier C Use of calibrated automated thrombinography +/− thrombomodulin to recognise the prothrombotic phenotype Thromb Haemost 2006 96 05 562 567 17080211

[35] Calabro P Yeh E T Obesity, inflammation, and vascular disease: the role of the adipose tissue as an endocrine organ Subcell Biochem 2007 42 63 91 17612046

[36] Pischon T Girman C J Hotamisligil G S Rifai N Hu F B Rimm E B Plasma adiponectin levels and risk of myocardial infarction in men JAMA 2004 291 14 1730 1737 15082700 10.1001/jama.291.14.1730

[37] Lemkes B A Hermanides J Devries J H Holleman F Meijers J C Hoekstra J B Hyperglycemia: a prothrombotic factor? J Thromb Haemost 2010 8 08 1663 1669 20492456 10.1111/j.1538-7836.2010.03910.x

